# The challenge of education for sustainability in higher education: key themes and competences within the University of the Basque Country

**DOI:** 10.3389/fpsyg.2023.1158636

**Published:** 2023-07-03

**Authors:** Nahia Idoiaga Mondragon, Ion Yarritu, Estibaliz Saez de Cámara, Nekane Beloki, Laura Vozmediano

**Affiliations:** ^1^Department of Developmental and Educational Psychology, Faculty of Education of Bilbao, University of the Basque Country, Bilbao, Spain; ^2^Department of Chemical and Environmental Engineering, School of Engineering of Bilbao, University of the Basque Country, Bilbao, Spain; ^3^Department of Sociology and Social Work, Faculty of Education of Bilbao, University of the Basque Country, Bilbao, Spain; ^4^Department of Social Psychology, Faculty of Psychology, University of the Basque Country, Bilbao, Spain

**Keywords:** higher education, sustainability, competences, university teachers, education for sustainability, SDG

## Abstract

**Introduction:**

One of the major challenges for higher education institutions in the last decade has been (and will continue to be) the integration of sustainability into their curricula and the development of sustainability competences in students. Education for Sustainability (ES) can help prepare students to meet the challenges of making societies more sustainable. However, as a first step toward this goal, teachers need to incorporate ES into their teaching. In this regard, this research aimed to analyze if members of the teaching staff have started this integration and, if so, which sustainability-related topics have been introduced and which skills do they consider contribute to the development of future graduates.

**Methods:**

A questionnaire was administered to teaching staff at the University of the Basque Country in 2022. A total of 403 teachers completed the questionnaire, expressing their perceptions through open-ended questions.

**Results:**

In general terms teachers incorporate ES into their teaching (71.22%). However, they do this mainly within the framework of two general themes: “Environmental awareness and energy” – most used by teachers of experimental sciences and engineering – and “Social commitment,” most used by teachers of social sciences and those who are familiar with the UN 2030 Agenda for Sustainable Development. Regarding the key competences that ES provides for future graduates, those most frequently mentioned were “training of professionals committed to society” and “critical thinking and ethics.” These competences were particularly notable in the discourse of teachers who were aware of the 2030 Agenda and who use active methodologies in their classrooms. Finally, the opinion that sustainability has little to do with their teaching (28.78%) was notably expressed by teachers less familiar with the 2030 Agenda.

**Discussion:**

Thus, it can be concluded that, aside from knowledge of the 2030 Agenda, factors such as the sustainability policy of the institution, area of teaching expertise, and the use of active methodologies all play a significant role in determining whether competences for sustainable development are integrated into higher education teaching.

## Background

In September 2015, world leaders at the United Nations (UN) General Assembly unanimously approved the “Transforming Our World: the 2030 Agenda for Sustainable Development” (United Nations, 2015), one of the most ambitious and relevant global agreements in recent decades. At the heart of this agenda are 17 Sustainable Development Goals (SDGs), categorized into 5 Ps (Planet, People, Prosperity, Peace, and Partnerships), with 169 targets aimed at guiding all countries toward collaboratively solving our world’s most demanding challenges by 2030. These challenges include ending poverty and hunger; preserving natural resources and protecting ecosystems from degradation; addressing climate change; ensuring that all people can enjoy prosperous, healthy, and fulfilling lives; and fostering peaceful, just, and inclusive societies free from fear and violence.

The complex challenges covered by this agenda have never been more important and urgent. With just half of the time remaining to the 2030 deadline for achieving the SDGs, a growing understanding of the urgent need to address climate change (IPCC, 2021), the post-COVID-19 crisis, and increasing levels of violence and wars showcase the interconnections between our environment, prosperity, and well-being. The pursuit of SDGs requires deep transformations in how societies and economies function and how we interact with our planet. At the same time, these transformations toward economic development that is socially inclusive and environmentally sustainable require all sectors and agents to operate in more collaborative, interconnected, systemic, and committed ways (Sachs et al., 2019).

Universities and Higher Education Institutions play a unique and significant role in helping society achieve SDGs through their research, teaching and learning, campus operations, and leadership (UNESCO MGIEP, 2017; Serrate González et al., 2019; Ferguson and Roofe, 2020; Ruiz-Mallén and Heras, 2020). The role of these institutions in promoting enlightenment, humanism, and prosperity of societies is of immense importance, and as such, they have historically changed societies for the better (Pinker, 2011). Moreover, according to SDSN (2020), none of the SDGs will be fully achieved without the contribution of academia.

One of the most important ways in which universities can contribute to SDGs is to provide Education for Sustainability (ES). The SDGs themselves recognize the importance of creating the knowledge, skills, and mindsets that can enable different sectors (and learners in general) to achieve the SDGs. This idea has been explicitly expressed in the form of several targets, such as SDG 4.7, which states that: “By 2030, ensure that all learners acquire the knowledge and skills needed to promote sustainable development.”

ES in Higher Education gained attention after the approval of the 2030 Agenda for Sustainable Development, and it is considered one of the contemporary indicators of educational quality (Anghel and Neculau, 2022). Moreover, ES in higher education “aims to encourage young people to become active participants in building more sustainable societies” (Finnveden et al., 2020). To achieve the latter, ES tries to help students develop the necessary knowledge, competences, and mindsets to tackle the complex challenges of Sustainable Development through whichever career or life path they take. These include: (1) a general understanding of Sustainable Development and SDGs; (2) crosscutting skills to make sense of complex challenges and to devise and implement solutions; (3) specific knowledge and skills that enable each profession to contribute to the SDGs and, (4) mindsets that promote positive societal change (SDSN, 2020). Wiek et al. (2011) synthesized the literature on key sustainability competencies based on competence as problem-solving capacity; they conclude that these are systems-thinking, anticipatory, strategic, normative and interpersonal competences. These competences are can guide the design of programs and courses in sustainability and teaching and learning evaluations in higher education, but they highlight that they require adaptation. More recently, Brundiers et al. (2021), based on the previous work, conducted a Delphi study with international experts in sustainability education. They added two additional competences (intrapersonal and implementation competences) and proposed a hierarchy of competencies, values-thinking competency as underpinning competency. This study also revealed that competencies are not naturally developed in teaching–learning settings and that their development requires deep transformations.

To achieve all of the above, universities need to scale up their existing education programs and implement new initiatives that go beyond “business as usual.” These comprehensive programs should enable students to become equipped with the competences necessary for sustainable development at each level of education (undergraduate degree, master courses, Ph.D., and executive training). Accordingly, universities need to develop new transformative teaching-learning activities to train students to think systematically about the major challenges (e.g., climate change, resource depletion, loss of biodiversity, poverty, hunger, and violence) from several disciplinary perspectives, with solution-oriented (action-based) learning, and multi-actor involvement, none of which are part of current standard practice within universities (González-Pérez and Ramírez-Montoya, 2022).

Since 2015, there has been a growing recognition of the importance of sustainable development and SDGs for all students in our increasingly complex 21st century and the exceptional potential of universities to deliver global sustainability. Nonetheless, there is still a long way to go before this sector delivers on its full potential to achieve the SDGs. One of the areas where there is a particular need to accelerate action is the delivery of ES. ES is crucial in promoting a “crosscutting” understanding of key sustainable development issues and the corresponding skills and key competences required by students and future “implementers” of Sustainable Development (UNESCO, 2017). The latter entails understanding the concept of sustainable development and related ideas, such as human rights, social justice, planetary boundaries, models of nature-society-economy interactions and dependencies, diversity, gender equality, sustainability, global citizenship, and inequality. Meeting these targets also requires knowledge of the key global and local sustainable development challenges, such as climate change and inequality, and their causes, dynamics, and interconnections. Competences that are key to the general education of all learners in addressing the SDGs are systems thinking, critical thinking, self-awareness, reflection, integrated problem-solving, and anticipatory, normative, strategic, and collaboration competences; creativity; entrepreneurship; curiosity and learning skills; human-centered design thinking; social responsibility; partnership competences; interdisciplinarity skills; critical-ethical analytical skills; influencing change; behavioral insights; cross-cultural skills; empathy; and communication (SDSN, 2020). Recently, the European Sustainability Competence Framework, known as GreenComp, organized these competences into four interrelated areas: (1)“embodying sustainability values,” including the competences (a) valuing sustainability, (b) supporting fairness and, (c) promoting nature; (2) “embracing complexity in sustainability,” including (a) systemic thinking, (b) critical thinking, and (c) problem framing; (3) “envisioning sustainable futures,” including (a) futures literacy, (b) adaptability, and (c) exploratory thinking; and (4) “acting for sustainability,” including (a) political agency, (b) collective action and, (c) individual initiative (Bianchi et al., 2022).

ES can take a wide range of forms within a university as it includes several distinct elements, and these can be implemented in a variety of ways, at varying levels and degrees of depth (macro, meso, and micro) and delivered to a wide range of potential students (Fia et al., 2022). These options vary from developing new courses, programs, or initiatives that focus specifically on the SDGs along with the knowledge, competences, and mindsets needed to implement these objectives to integrating relevant elements of ES into the existing curriculum across all relevant disciplines (UNESCO MGIEP, 2017). Unfortunately, there is no ready-made formula for such success; each institution must find the combination of approaches and ways that best suit their particular circumstances.

The integration of elements of ES (action-based learning, transdisciplinarity, and multi-agent collaboration) is challenging since this entails a seismic shift from how teaching and learning are currently organized and delivered (Binyamin and Ben Slama, 2022). Indeed, such changes require the support, collaboration, and involvement of the teaching staff, since faculty is integral to curriculum and educational transformation (UNESCO, 2022). Nevertheless, for a variety of reasons (e.g., resistance to change, not seeing the relevance of ES, lack of knowledge of the 2030 Agenda, lack of access to appropriate resources, or lack of skills in innovative methods), teaching staff may not be able to provide this support and collaboration.

This study aims to determine the ways in which university teachers are currently engaging with the ES space and their perception of the utility and importance of ES for future graduates. Although this work is focused on the University of the Basque Country (UPV/EHU) with the aim of maximizing the contribution of this institution to the 2023 agenda, our ultimate goal is to identify priorities, opportunities, and gaps for gradually integrating elements of ES into the wider higher educational framework.

### Case study: University of the Basque Country (UPV/EHU). EHU Agenda 2030 for sustainable development and IKD i^3^ strategy

UPV/EHU is a public research university deeply rooted in Basque society, open to the world, and driven by intellectual leadership with a strong ethical and social commitment. With 20 schools and faculties distributed over three campuses (Araba, Bizkaia, and Gipuzkoa), the UPV/EHU offers a wide range of degrees and courses in all knowledge areas from experimental sciences, engineering, and humanities to health sciences, economics, fine arts, and architecture. The university offers 103 undergraduate programs and 174 postgraduate programs, all of which are fully adapted to the European Higher Education System. It has a total undergraduate and postgraduate enrolment of 44,000 students, more than 4,000 teaching staff, and 1,500 administrative staff.

Like many other Higher Education Institutions, the UPV/EHU has considered the 2030 Agenda and the SDGs as a framework that can be accommodated the vast number of programs implemented in recent years. Consequently, in 2017, the university embarked on a reflection process to define a strategy aimed at bringing the work of the University in line with SDGs and to move toward making a verifiable, pragmatic contribution. As a result of this process, a plan was put into place, entitled “EHU Agenda 2030 for Sustainable Development” (UPV/EHU, 2019a). This strategy describes the UPV/EHU’s proposed contribution to 12 of the 17 SDGs, with the addition of its commitment to linguistic and cultural diversity (SDG 17 + 1) and the sectoral plans comprising the Equality Campus, Inclusion Campus, and Planet Campus. Additionally, the plan includes a panel of indicators for sustainable development, which addresses the technical aspects of monitoring SDGs and the selected targets.

**Figure 1:**
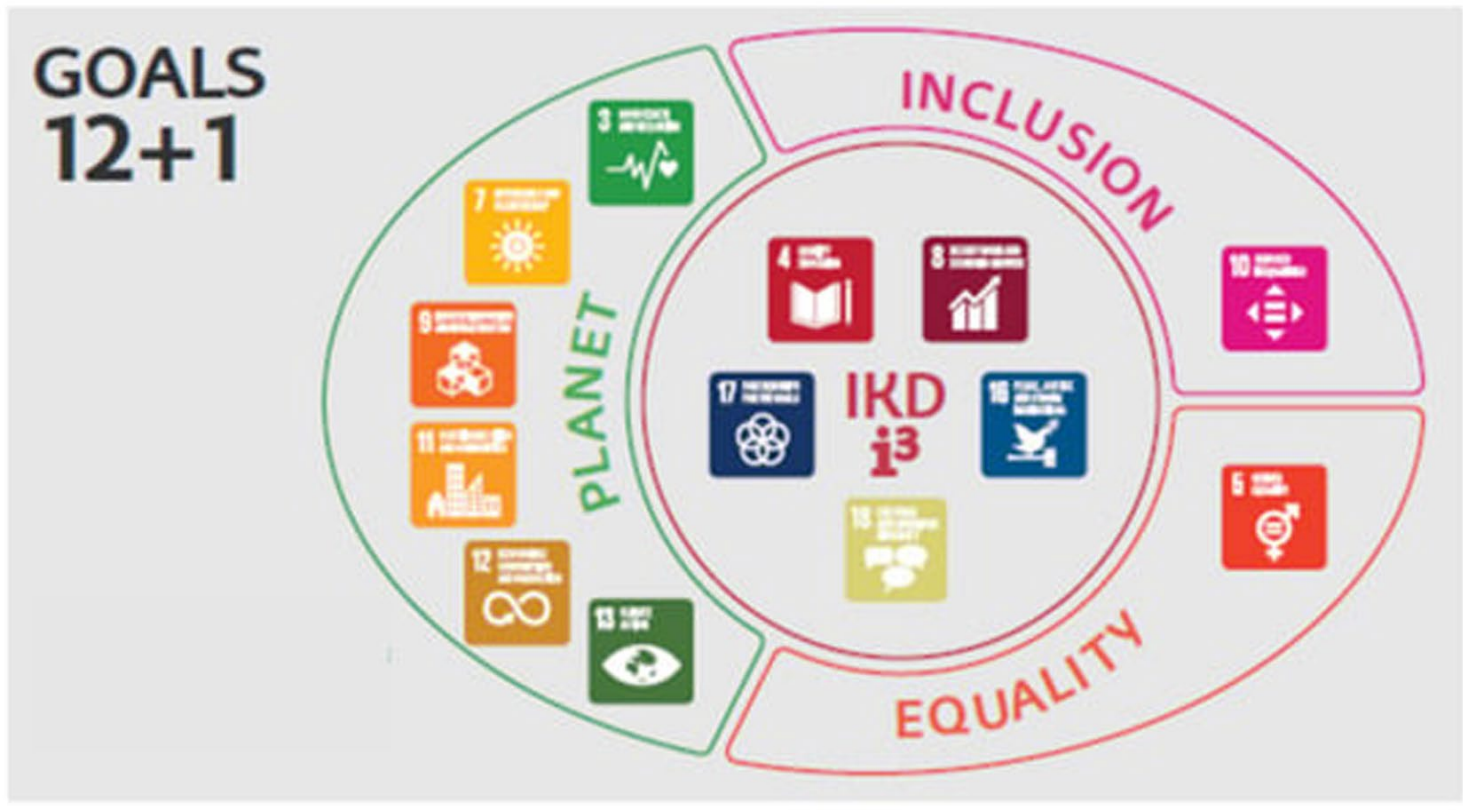
Concentric circle diagram to depict the EHU Agenda 2030 for sustainable development. Source: UPV/EHU (2019a). Reproduced with permission from University Of the Basque Country.

One of the most important aspects of this strategy is concentric logic (depicted in Figure 1), which promotes a common interdependent relationship, particularly in the teaching-learning processes, aimed at overcoming excessively isolated and fragmented visions and working methods. The main aim (core) of university activity is SDG 4, which highlights all the teaching-learning processes in their broadest and most comprehensive forms. This matrix includes SDG 8, which strives for employability and the contribution of university education to sustainable economic development (SDG 16), covering every aspect of education for human rights as an essential component of curricular logic, while SDG 17 is addressed by incorporating the entire spectrum of cooperation for development, commitment, and social transfer, alongside SDG 17 + 1. In addition, three sectoral plans were deployed alongside this core: the Gender Equality plan, centered on SDG 5; the Inclusion plan, which contributes to SDG 10, and the Health and Environmental Management Plan, covering SDG 3, SDG 7, SDG 9, SDG 11, SDG 12 and SDG 13.

In addition, UPV/EHU’s educational model IKD (UPV/EHU, 2010) underwent a review of this process to bring it into line with modern pedagogical trends, resulting in the renewed European Commission (2017) and its funding programs (Erasmus+, Marie Sklodowska-Curie Actions and Horizon 2020, among others) together with the 2030 Agenda for Sustainable Development. The outcome was IKD i^3^, which translates to multiplying learning (“ikaskuntza” in Basque) by research (“ikerkuntza”) and sustainability (“iraunkortasuna”), namely the exponential growth of each of these terms (i.e., to the power of three), paving the way for processes and products hitherto unknown.

In parallel, the UPV/EHU’s Catalogue of Transversal Competences was published, setting out eight competences common to all UPV/EHU degree qualifications (UPV/EHU, 2019b). These competences are Autonomy and Self-regulation, Social Commitment, Communication and Plurilingualism, Ethics and Professional Responsibility, Information Management, Digital Citizenship, Innovation, and Entrepreneurship, Critical thinking, and Teamwork.

### Research gaps and objectives

Given the preceding theoretical framework, there is a notable gap or lack of knowledge on whether university teachers are integrating sustainability and the SDGs of the 2030 Agenda into their daily teaching practice and, if so, how – and what – are the competences acquired by future graduates. This lack of knowledge is concerning, as these teachers are critical agents in implementing ES and initiatives that shape the transformation of educational content, approaches, and methods (Olaskoaga-Larrauri et al., 2021). Moreover, to the best of our knowledge, no study has freely gathered the voices of the teaching staff directly regarding the sustainability topics and competences covered in their lectures. Therefore, this research aims to answer the following two research questions: (1) Which sustainability-related issues and SDGs of the 2030 Agenda are UPV/EHU teachers incorporating into their daily teaching? (2) Which competences or skills do teachers believe that ES provides for their future graduates? Based on these questions, the fundamental objective of this research was to explore which sustainability-related topics and competences are being incorporated by UPV/EHU into their courses.

## Methods

### Participants and questionnaire

The University of the Basque Country (UPV/EHU) comprises 4,314 teachers (excluding research-only staff and visitors). Participants were recruited from April to June 2022. The University’s conventional notification systems (including mailing, notice boards, and social networks) were used to distribute information about the project and provide the link to the questionnaire. First, the information stated that participation was voluntary and anonymous, and that the questionnaire could be completed in 15 min, both in Basque and Spanish. Second, participants were informed that this research has the approval of the Ethics Committee of the UPV/EHU (Ref.: M10/2022/107). In this introduction, participants had to agree to participate in the study by answering YES/NO, with A NO answer ending the questionnaire. Finally, a total of 403 teachers responded to the questionnaire, a significant sample considering the size of the population and, assuming a confidence level of 95%, this ensured that the results can be extrapolated to the population with a margin of error of less than 5% (4.65%).

The questionnaire was composed of close-ended questions on sociodemographic variables and teaching activity, and open-ended questions regarding their practice and perception of ES. First, information was gathered on gender and age, along with data related to teaching activity, including field of teaching, experience participating in educational innovation projects (EIP), knowledge of the UN 2030 Agenda, perception of the importance of competences for sustainability for graduates, knowledge of UPV/EHU’s IKDi^3^ strategy, and experience applying active methodologies in teaching. Second, participants had to answer the following open-ended questions regarding their practice and perception of ES: (Q1) Do you include any themes related to sustainability or SDGs in your courses? Which ones? Do you explicitly relate these to sustainability? (Q2) What do you think sustainability or Education for Sustainability brings to graduates of the degrees you teach? The teachers could answer freely by expanding on their responses to these two questions as much as they wished.

### Data analysis method

Closed-ended questions were analyzed using SPSS v.26 software for data analysis, performing descriptive analyses, whereas the responses to the open questions were analyzed using Iramuteq software for lexical analysis (Ratinaud, 2009; Ratinaud and Marchand, 2012) and specifically the Reinert method for corpus analysis (Reinert, 1983, 1990). This method has frequently been used for the study of open questions (Souza et al., 2018; Larruzea-Urkixo et al., 2021; Idoiaga Mondragon et al., 2022; Legorburu et al., 2022), and it has been chosen because it eliminates the usual reliability and validity problems in text analysis (Klein and Licata, 2003). Using this method, which follows a descending hierarchical cluster analysis format, a series of classes and statistical cues in the form of typical words and text segments are obtained (Idoiaga and Belasko, 2019). Specifically, the software identifies the words and text segments with the highest Chi-square values, that is, those that best identify each class or idea that the participants have repeatedly mentioned.

Following previous research using the Reinert method (Camargo and Bousfield, 2009), the raw data were introduced into the Iramuteq software, and the most significant items of vocabulary in each class were selected based on the following three criteria: (1) an expected value of the word greater than 3; (2) evidence of an association based on the Chi-square statistic, tested against the class (*χ*^2^ ≥ 3.89 (*p* = 0.05); *df* = 1) and; (3) the word appearing mainly in that class with a frequency of 50% or more. Iramuteq software also determined which text segments were associated with each class or group of words and classified them according to their chi-square value. This study collected text segments with the most significant chi-squares of each class.

Once these “lexical universes” were identified, they were associated with “passive” variables (independent variables). In the present case, the passive variables were the teaching area of the participants, involvement in Educational Innovation Projects (EIP), knowledge of the 2030 Agenda for Sustainable Development, awareness of the importance of competences for sustainability for graduates, knowledge of the UPV/EHU’s IKDi^3^ strategy, use of active methodologies in teaching, and gender. As a result, we obtained a series of classes composed of typical words and text segments (quotations) with the highest chi-square values. The total chi-square value of each quotation is the sum of the chi-square values of each word in that quotation concerning the class. This value provides the basis for “interpreting” the classes as lexical universes. The Reinert method produces statistical, transparent, and reproducible data until the final point of interpretation, where the analyst then assigns a label. Finally, the researchers will give a title to the group of words and text segments clustered by the software (Schonhardt-Bailey, 2013). In this final phase, this study employed a systematic process to create the labels or titles of each class. In that process, two researchers independently named each class based on the words and associated quotations, after which a third researcher created a final label that was approved by all three.

## Results

The results were analyzed in two phases. First, descriptive analyses of the closed questions were conducted to obtain a complete picture of the responses to the questionnaire. Second, lexical analysis was conducted on the responses to the open-ended questions.

Analyses of responses to the closed-ended questions revealed that 230 of the 403 respondents were women (57.1%), 168 men (41.7%), and five were non-binary (1.2%). Furthermore, regarding age, 0.5% were under 30 years old, 17.9% were between 31 and 40 years old, 35.2% were between 41 and 50 years old, 36.7% were between 51 and 60 years old, and 9.7% were over 60 years old. Concerning work experience, 20.8% have worked at the UPV/EHU for less than 10 years, 35.5% between 10 and 20 years, 25.1% between 21 and 30 years, and 18.6% more than 30 years. Finally, regarding the teachers’ field of expertise, 34% were in social and legal sciences, 24.6% in engineering and architecture, 15.1% in experimental sciences, 14.6% in health sciences, and 11.7% in arts and humanities.

Next, a lexical analysis was conducted to identify the main ways in which teachers embed sustainability in their classes and the related competences acquired by their future graduates. The complete corpus contained 15,731 words, of which 2,103 were unique. The top-down hierarchical analysis of the Reinert method divided the corpus into 888 segments and five classes. A class is each reason or idea represented by a set of typical words and text segments. Four of the classes start with the recognition of incorporating sustainability into teaching. In particular, the first two classes describe the topics teachers integrate into their lectures to work on with their students, which were labeled “Environmental awareness and energy” and “Social Commitment. “The next two classes present the competences offered by ES to the graduates of their degree courses, and these were labeled “Professionals committed to society” and “Critical thinking and ethics.” Finally, the fifth class refers to the failure to recognize the importance of integrating sustainability into teaching and was labeled “Sustainability not relevant.” The results of this analysis are shown in Figure 1.

The sustainability theme most frequently described as being integrated into teaching was “Environmental awareness and energy” (22.73%). Within this class, the teachers state that they primarily cover topics linked to the environment, use of energy and materials, reduction of consumption, or growth. These topics are central to SDG 7 (Affordable and clean energy), SDG 6 (Clean water and sanitation), SDG 12 (Responsible production and consumption), and SDG 13 (Climate action). This class was mentioned significantly more by teachers in experimental sciences (*p* < 0.05) and engineering and architecture (*p* < 0.05). To provide a context for these words, the characteristic text segments or quotations of the class were examined. The following are some of the most significant text segments of this class:

- “Clean water and sanitation; available and clean energy; climate action; life in terrestrial ecosystems. Today there is a lot of work in geology from the point of view of contaminated land and the environment” (*χ*^2^ = 214.29; Experimental sciences, male, less than ten years worked).
- “SDG 6 clean water and sanitation; SDG 7 affordable and clean energy. In environmental engineering, these are essential tools for developing environmental projects. Therefore, we work on knowledge of technologies to avoid or minimize environmental pollution in the three media: air, water, and land” (*χ*^2^ = 274.95; Engineering and architecture, female, less than ten years worked).
- “SDG 12 responsible production and consumption. We work on good practices to be applied in their professional field, in addition to being environmentally friendly, which result in a better use of finite natural resources” (*χ*^2^ = 235.47; Health sciences, female, between 21 and 30 years worked).

This class was followed by “Social commitment,” which emerged with a weight of 13.11%. The responses in this class also refer to issues relevant to the SDGs but with a more social focus, such as SDG 4 (Quality Education), SDG 5 (Gender equity), SDG 3 (health and well-being), SDG 16 (peace, justice, and strong institutions), SDG 10 (reducing inequalities) and SDG 17 (partnerships for achieving the goals). This class was significantly more mentioned by teachers in social sciences and law (*p* < 0.01), particularly those who had participated in EIP (*p* < 0.01), are aware of the UPV/EHU’s IKD i^3^ strategy (*p* < 0.01) and the 2030 Agenda (*p* < 0.01) and who believe that sustainability is important for the competences of their graduates (*p* < 0.01). The following are some of the most significant text segments from this class:

- “Quality education (SDG 3), health and well-being (SDG 5), gender equality (SDG 17) partnerships for achieving the goals (SDG 10), reducing inequalities (SDG 10). It provides a different point of view from which to approach holistic education” (*χ*^2^ = 569.40; Social and legal sciences, female, 10–20 years worked).
- “Within the SDGs in my subjects the ones I develop the most are SDG 4 quality education SDG 5 gender equality SDG 10 reduction of inequalities SDG 16 peace justice and strong institutions” (*χ*^2^ = 478. 30; Social and legal sciences, female, over 30 years worked).
- “We always work on health and well-being, quality education, gender equality and responsible production and consumption. All this together with the ability to make decisions and implement effective actions and also unique to transversal competences” (*χ*^2^ = 469.85; Social and legal sciences, female, 10–21 years worked).

Regarding beliefs about the sustainability competences acquired by future graduates, the third class emerged, labeled “Professionals committed to society,” with a weight of 20.51%. This refers to the way in which these competences could help future graduates shape the world of work or companies and foster a society with a more collective and global vision in future decision-making. This class was significantly more frequently mentioned by teachers who are aware of the 2030 Agenda (*p* < 0.05), who believe that sustainability is important for the competences of their graduates (*p* < 0.05), and who use active methodologies in their teaching (*p* < 0.05). The most significant text segments in this class are:

- “Graduates will have a vision of business management not only based on the generation of business profits, but also on the need to seek social benefit and the well-being of workers, consumers and society in general” (*χ*^2^ = 92. 69; Social and legal sciences, male, 21–30 years worked).
- “Feminism, anti-racism, non-discrimination against vulnerable people and groups… all this will allow them to be more professional and to be able to make a positive contribution to society in general, from a position of responsibility such as in the field of communication” (*χ*^2^ = 129.48; Social and legal sciences, female, less than 10 years worked).
- “I believe that sustainability will be increasingly present in our daily lives. Also, in the world of work. And education for sustainability will provide graduates with the tools not only to respond to the demands of their workplaces but also to achieve a more sustainable and fairer world” (*χ*^2^ = 109.34; Health sciences, female, less than 10 years worked).

The second most frequent (18.80%) competence expressed by teachers was “Critical thinking and ethics.” In this regard, the teachers describe the importance of critical thinking and ethics for future professionals, which are skills that students can develop through current active projects. This competence was significantly more mentioned by teachers who are aware of the 2030 Agenda (*p* < 0.01), who believe that sustainability is important for the competences of their graduates (*p* < 0.001), women (*p* < 0.05) and who use active methodologies in their teaching (*p* < 0.05). The following are the most significant text segments of this class:

- “Now they know what it is and how it can be applied to their professional and personal environment. Having a critical point of view with technical projects, both in terms of caring for the planet, as well as social needs and the economic development model, is essential” (*χ*^2^ = 162.69; Engineering and architecture, female, between 21 and 30 years worked).
- “Yes, the students carry out a group project in which they deal with the sustainable development objectives they consider from the perspective of the subject. This work is fundamental for their future critical, ethical, and proactive professional practice in today’s society. Since as graduates they can play a decisive role in achieving the objectives of sustainable development” (*χ*^2^ = 143.83; Social and legal sciences, female, 21–30 years worked).
- “Having undergone active training that provides them with a fusion of technical and transversal competences with which they can see the reality of their professional thematic area differently. In a transformative way, they will become aware of critical thinking and professional ethics” (*χ*^2^ = 110.96; Engineering and architecture, female, 21–30 years worked).

Finally, the fifth class emerged with a weight of 28.78%. This class includes the responses from some teachers explaining why they do not cover sustainability in their courses and therefore do not provide their graduates with these competences. This class was called “Sustainability not relevant,” as it indicated that sustainability is of no importance to the course being taught or that it was a fad that was assigned too much importance. This class was significantly more frequently mentioned by teachers who are not aware of the 2030 Agenda (*p* < 0.05), those from the field of health sciences (*p* < 0.01), those who believed that sustainability competences are unimportant for their future graduates (*p* < 0.001) and by men (*p* < 0.001). The following are some of the most significant responses from this class:

- “My subject has nothing to do with sustainability or sustainable development. I have heard many times that it is important, I think it is a fashion, but in the subjects, I teach I do not see anything related to this topic” (*χ*^2^ = 250.06; Arts and humanities, male, between 10 and 20 years worked).
- “It seems to me that it is not such an important issue when we have so many challenges to address in terms of training our future graduates. It is a fashionable subject, but it needs to be addressed in a crosscutting way and it is very complicated and forced depending on the type of subject taught” (*χ*^2^ = 186.14; Health sciences, male, 10–20 years worked).
- “The concepts of mechanical analysis and design are alien to sustainability. It is their application in the industrial environment that is related to this aspect, and it is an issue that is not and should not be dealt with in the subjects I teach” (*χ*^2^ = 156.12; Engineering and architecture, male, less than 10 years worked).

To summarize, Table 1 shows the main findings of this research.

**Table 1:** Main research findings.

Sustainability		Class title	Weight	This class was significantly more frequently mentioned by
Incorporates sustainability into their teaching	Sustainability-related issues incorporated	Class 1: Environmental awareness and energy	22.73%	Teachers from experimental sciences (*p* < 0.05) Teachers from engineering and architecture (*p* < 0.05)
		Class 2: Social commitment	13.11%	Teachers from social and legal sciences (*p* < 0.01) Teachers who have participated in EIP (*p* < 0.01) Teachers who are aware of the UPV/EHU’s IKD i^3^ strategy (*p* < 0.01) and the 2030 Agenda (*p* < 0.01) Teachers who believe that sustainability competences are important for their future graduates (*p* < 0.01)
	Competences acquired by graduates through ES	Class 3: Professionals committed to society	20.51%	Teachers who are aware of the 2030 Agenda (*p* < 0.05) Teachers who believe that sustainability competences are important for their future graduates (*p* < 0.05) Teachers who use active methodologies in their teaching (*p* < 0.05)
		Class 4: Critical thinking and ethics	18.80%	Teachers who are aware of the 2030 Agenda (*p* < 0.01) Teachers who believe that sustainability competences are important for their future graduates (*p* < 0.001) Women (*p* < 0.05) Teachers who use active methodologies in their teaching (*p* < 0.05)
Do not incorporate sustainability in their teaching		Class 5: Sustainability not relevant	28.78%	Teachers who are unaware of the 2030 Agenda (*p* < 0.05) Teachers from health sciences (*p* < 0.01) Teachers who believe that sustainability competences are not important for their future graduates (*p* < 0.001) Men (*p* < 0.001)

## Discussion

Quality Education is explicit in SDG 4 (Target 4.7) and is reflected in other goals and targets, such as the decent work SDG8 and the equality SDG5 (Shulla et al., 2020). Education for sustainability (ES) is therefore considered key to attaining these goals by promoting lifelong learning and the ability to make informed choices and take responsible action regarding environmental integrity, economic viability, and social justice for present and future generations while respecting cultural diversity (UNESCO, 2016; Rieckmann, 2018; Agbedahin, 2019; English and Carlsen, 2019; Evans, 2019). This research aimed to explore this framework by analyzing specifically what sustainability-related topics the teaching staff of UPV/EHU introduce in their courses and the competences they believe are consequently acquired by their future graduates.

Concerning the first research question, the results of the questionnaire reveal that, in general, teachers at the UPV/EHU integrate sustainability into their teaching, as promoted by this institution through its EHU Agenda 2030 for sustainable development and its educational strategy IKD i^3^ (UPV/EHU, 2019a). This result is unsurprising considering that one of the main facilitators of the integration of ES in university teaching is the perception of organizational support (Cotton et al., 2009; Cebrián et al., 2015). In particular, previous evidence suggests that the implementation of training programs for teachers in the field of sustainability – in line with the actions implemented within the IKDi3 framework – could be beneficial in this regard (Barth and Rieckmann, 2012; Vare et al., 2019; Scherak and Rieckmann, 2020).

The teaching staff of the UPV/EHU integrate sustainability by focusing on two main themes: “Environmental and energy awareness” and “Social commitment.” Concerning environmental and energy awareness, in 22.73% of the total responses, teachers state that they address at least the following SDGs in their lectures: affordable and clean energy (SDG 7, *χ*^2^ = 20.94), Clean water and sanitation (SDG 6, *χ*^2^ = 17.47), Responsible consumption and production (SDG 12, *χ*^2^ = 12.44), and Climate Action (SDG 13, *χ*^2^ = 12.44). All these SDGs are included in the EHU Agenda 2030 and its educational strategy IKD i^3^ (UPV/EHU, 2019a; Sáez de Cámara et al., 2021). These findings seem to demonstrate the impact of this educational model on the way in which the teaching staff of UPV/EHU approach their professional practice.

The theme of “Environmental and energy awareness” was most frequently mentioned by teachers of engineering, architecture, and experimental sciences (Watson et al., 2013; Leal Filho et al., 2019). This is closely aligned with an international trend where aspects related to climate change, energy crisis, sustainable cities, and water are predominant in studies of experimental sciences (Leal Filho et al., 2019) and engineering (Alexa et al., 2020). However, while the environment is one of the pillars of sustainability (Menon and Suresh, 2020; Zacchia et al., 2022), ES goes beyond this reductionist representation of sustainability and aims to foster a more holistic approach (Morrison-Saunders et al., 2022).

**Figure 2:**
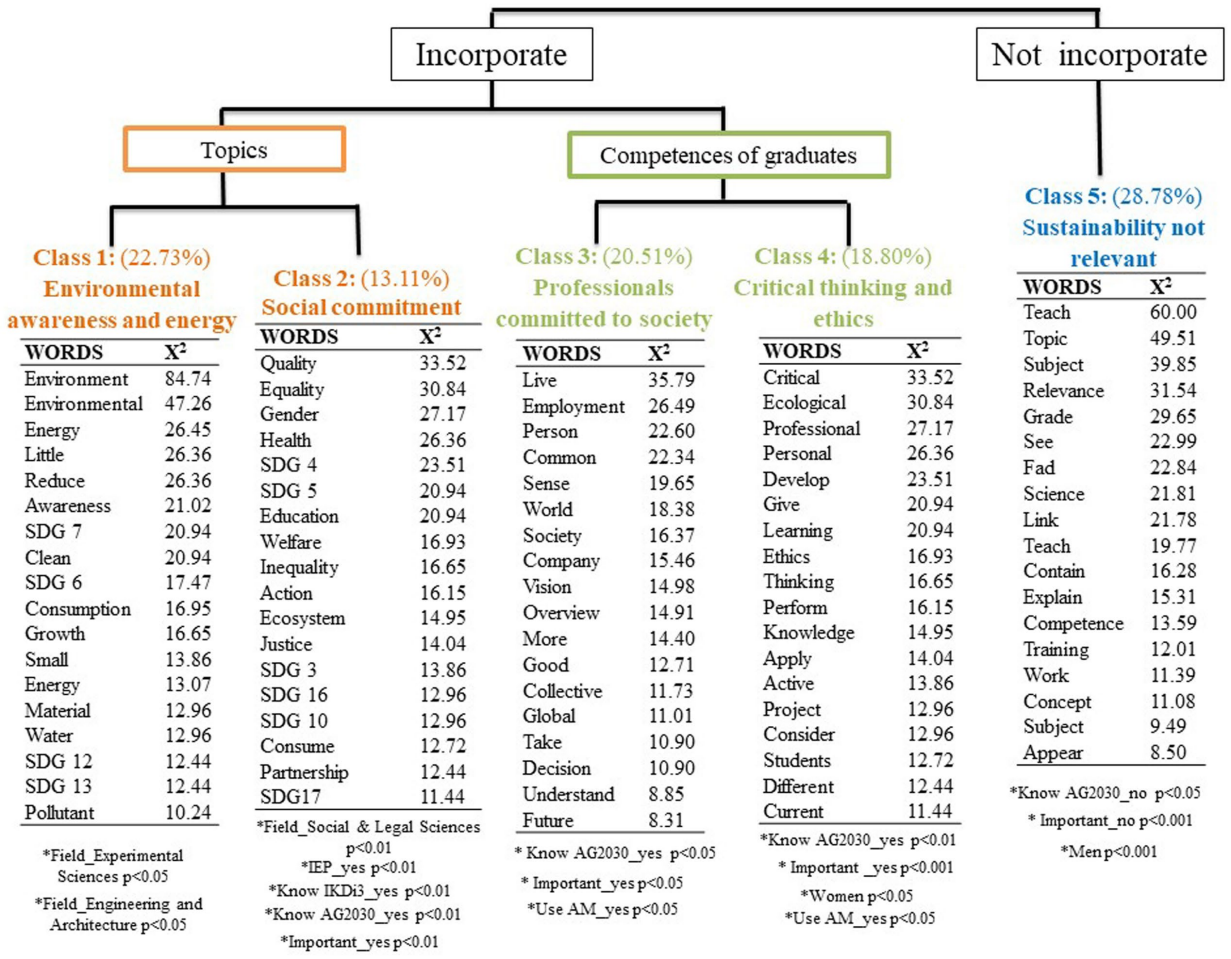
Hierarchical clustering dendrogram of the corpus extracted from the questionnaire’s response corpus, showing the words with the highest association for each class *χ*^2^ (1), *p* < 0.001, extracted by the Reinert method.

Concerning “Social commitment,” reference to this theme was made by teachers who integrate quality education (SDG 4, *χ*^2^ = 23.51), gender equality (SDG 5; *χ*^2^ = 20.94), Good health and well-being (SDG 3, *χ*^2^ = 13.86), peace, justice, and strong institutions (SDG 16; *χ*^2^ = 12.96), reduced inequalities (SDG 10; *χ*^2^ = 12.96) and partnerships for the goals (SDG 17; *χ*^2^ = 11.44) into their teaching. Moreover, it is worth noting that teachers of social and legal sciences more frequently referred to these topics, as already observed in previous research studies (Uitto and Saloranta, 2017; Leal Filho et al., 2019; Ruiz-Morales et al., 2021). Three of these SDGs (SDG 4, SDG 16, and SDG 17) form the core of the IKD i^3^ strategy within the concentric logic proposed in the EHU agenda 2030 (Sáez de Cámara et al., 2021), depicted in Figure 2. The other three SDGs correspond to one of the three sectoral levels. For example, SDG 3 belongs to the planet campus already mentioned, SDG 5 represents the equity campus, and SDG 10 represents the inclusion campus. Furthermore, the integration of social commitment into teaching was more frequently mentioned by teachers who are aware of the 2030 Agenda and IKD i^3^ strategy, those who had participated in educational innovation projects, and those who believe that sustainability competences are important for future graduates. Thus, even though the incorporation of the sociocultural sphere into the definition of sustainability is more recent (Eizenberg and Jabareen, 2017; López et al., 2018; Grum and Kobal Grum, 2020), those teachers who are familiar with the 2030 Agenda and its incorporation into the higher education framework can integrate the social commitment dimension of sustainability into their teaching (Vale et al., 2022).

The laudable efforts of the United Nations with the 2030 Agenda (Torrent-Sellens, 2021) have made it possible to make visible, at least in part, the relationships between the three dimensions of sustainability (Purvis et al., 2019). While two of these three dimensions were very apparent in the responses of our teaching staff, the third, the economic dimension, did not emerge so clearly. Even if some references are made to the third dimension, this does not appear to be integrated into the curricula, unlike the environmental or sociocultural dimensions. This finding should be analyzed in more depth, although it may be the case that, as some authors point out, this dimension has been integrated into the other two (Sammalisto and Lindhqvist, 2008). However, it is also plausible that the participants had less pressing concerns about the economic dimension compared with other issues, which might explain why this dimension was mostly mentioned by those teachers working in this area of expertise (Sánchez-Carracedo et al., 2021). Whatever the reason, it would be interesting for the University to conduct a specific analysis of this dimension.

Regarding the second research question, which concerns the competences believed to be acquired by future graduates of the UPV/EHU through ES, our participants highlighted “Critical thinking and ethics,” together with “training of professionals committed to society” – both of which are key for adopting competency-based ES (Lozano et al., 2022) and are among the main values of universities according to UNESCO (2022). Moreover, a recent systematic review of teacher competencies around ES has identified critical thinking, community engagement, and connection as the skills needed by teachers to address current sustainability challenges from a critical and transformative perspective (Corres et al., 2020). Perhaps unsurprisingly, for the teachers in our study, the sustainability competences believed to be acquired by future graduates are precisely among those skills that they themselves require to successfully address ES. Therefore, it appears that the UPV/EHU is responding to one of the main sustainability challenges, which is to ensure that ES forms part of the curriculum so that students have acquired, by 2030, the appropriate knowledge, skills, and mindsets to promote sustainable development (Figueiró et al., 2022). Such competences will play a pivotal role in promoting sustainable lifestyles, human rights, gender equality, a culture of peace and non-violence, and global citizenship while valuing cultural diversity and the contribution of cultures to sustainable development (Leicht et al., 2018 p. 25), themes that emerged in the teachers’ responses.

In this respect, research suggests that to develop a sustainability competence paradigm, it is necessary to promote the benefits of ES, the use of pedagogical approaches, and the development of competences. At the same time, challenges must be addressed to avoid creating a white elephant (Lozano et al., 2022). In our case, and in line with what is proposed by these authors, again, teachers who are aware of the 2030 Agenda and who recognize the importance of sustainability, along with those that use active methodologies in their teaching, are the ones who can see the value of ES for equipping their future graduates with key competences. Therefore, prior knowledge of ES and how to apply it in practice could be key to developing sustainability skills.

Furthermore, it is striking that women mentioned “Critical thinking and ethics” more frequently than men. This finding could be related to the high feminization of the social sciences, health sciences, and education teaching staff, where critical thinking skills feature more predominantly in lectures (Acuña-Salazar et al., 2022).

Finally, 28.78% of the surveyed teachers said they did not cover sustainability issues in their teaching. It is important to note that the majority of this group was unaware of the 2030 Agenda and did not believe that ES could provide their graduates with relevant skills. In this regard, it is worth mentioning previous research that has addressed the challenges and opportunities of involving academic staff in ES. For example, Cebrián et al. (2015) have found that, although university faculty are generally willing to engage in ES, the failure to fully grasp the significance of sustainability could constitute a barrier to embedding this concept into higher education. Therefore, it is possible that a lack of knowledge about sustainability could underlie the reluctance of those teachers who do not consider ES to be relevant. Consistent with this idea, several studies have already shown that awareness and training in sustainability lead to better and greater integration of sustainability in teaching (Lozano et al., 2015; Olaskoaga-Larrauri et al., 2021; Moreno-Pino et al., 2022) since such training would be expected to promote the belief that sustainability is important for the education of students (Ordóñez and Lorenz, 2019).

Finally, it is noteworthy that those respondents who do not integrate sustainability were mainly men. Cuttica et al. (2015) have shown that women participate more than men in activities related to lifelong learning, as well as other voluntary and unpaid activities. Therefore, it is possible that this difference could underlie the lower understanding of sustainability among men, which might explain why male teachers seem to be more reticent and critical when it comes to engaging with ES.

### Implications for practice and limitations

This research reinforces the value and utility of the UPV/EHU’s IKD i^3^ strategy for investing in ES among its faculty (Sáez de Cámara et al., 2021). More specifically, the teachers surveyed in this study are committed to the proposals for integrating sustainability into their teaching-learning processes and, as evident in the results of this study, combine ES with the use of active methodologies. Therefore, it appears that when ES is implemented in the curriculum through active teaching and learning strategies, it facilitates the development of the sustainability competences necessary for future citizens and professionals (Albareda-Tiana et al., 2019).

Another notable finding is that knowledge of the 2030 Agenda for Sustainable Development – combined with active participation in its implementation through teaching innovation or sustainability projects – is a central characteristic of teaching staff who are more strongly committed to this dimension.

Our findings highlight the need for universities to adopt an educational model aligned with their own version of the 2030 Agenda. But it is also essential to disseminate such a model and make every effort to provide faculty with adequate training and implementation opportunities based on a real and meaningful commitment.

Finally, it should be noted that the main limitation of this study concerns the sample. First, we cannot ignore the fact that UPV/EHU has been a pioneering university in integrating the UN Agenda 2030 into its strategic framework. Hence, the context in which the study has been conducted could limit the ability to generalize our results. Second, we should be mindful of the possible impact of self-selection bias, since participation was voluntary. Therefore, the sample may be biased in terms of the participants’ knowledge and understanding of the significance of the issue addressed. However, the presence of a critical current in the responses has revealed the existence of a certain diversity and plurality among our study participants. Allowing teachers to provide unrestricted answers using the free-response research methodology could also have been helpful in providing richer and more diverse data. Finally, two possible lines of future research could help overcome the present study’s limitations. First, it will be of interest to analyze the impact of incorporating Agenda 2030 on student learning outcomes, and second, it might be useful to conduct comparative studies with samples from different universities.

## Conclusion

In the last decade, many efforts have been directed toward developing competences in higher education to ensure that students are prepared to meet the challenges of making societies more sustainable (Žalėnienė and Pereira, 2021; Kioupi and Voulvoulis, 2022). However, it is clear that we still have a long way to go. This research first aimed to analyze whether university teachers have begun this integration and, if this is the case, identify which sustainability-related themes are covered in their teaching. In addition, we sought to establish which ES competences they consider to be of value in the development of our future graduates.

In this vein, we have shown that universities should focus future efforts on implementing the following action points, which we consider fundamental for the significant incorporation of ES into our degree courses:

- Universities should design and define their own educational models that incorporate ES, such as the IKD i^3^ model in the case of the UPV/EHU.
- Teachers should be trained and educated on key sustainability issues. In other words, having an educational model is not enough. It is essential to disseminate this among the teaching staff and design training strategies that enable them to incorporate ES in their teaching. To this end, it is also important that these training strategies are of an integrated and holistic nature. More specifically, these programs should include a basic component of sustainability-related content along with strategies to integrate ES into lectures through the use of active and student-centered teaching and learning methodologies that promote student engagement.
- There is a critical need to foster a holistic view of sustainability. Our results suggest that it is vital to understand the three dimensions of sustainability (environmental, economic, and sociocultural) in a holistic and integrated manner. To do so, it would be interesting to promote collaborative teaching with experts from other disciplines by comparing and sharing different ways of understanding the SDGs, which would help to eradicate the fragmented work perspectives linked to specific areas of knowledge.

In short, we believe that universities must continue to promote and support the integration of ES in their teaching models, strategies, and curricula, to be able to provide students – that is, our future professional citizens – with the key competences they will need to address present and future societal challenges.

## Data availability statement

The raw data supporting the conclusions of this article will be made available by the authors, without undue reservation.

## Ethics statement

The studies involving human participants were reviewed and approved by the Ethics Committee of the UPV/EHU (Ref.: M10/2022/107). The patients/participants provided their written informed consent to participate in this study.

## Author contributions

NI and IY designed the work, collected the data, and made a final approval of the version to be published. NI analyzed and interpreted the data. NI, ES, NB, and LV drafted the article. LV, NB, ES, and IY done a critical revision of the article. All authors contributed to the article and approved the submitted version.

## Conflict of interest

The authors declare that the research was conducted in the absence of any commercial or financial relationships that could be construed as a potential conflict of interest.

## Publisher’s note

All claims expressed in this article are solely those of the authors and do not necessarily represent those of their affiliated organizations, or those of the publisher, the editors and the reviewers. Any product that may be evaluated in this article, or claim that may be made by its manufacturer, is not guaranteed or endorsed by the publisher.
